# Willingness to wait covaries with endogenous variation in cortisol

**DOI:** 10.3389/fpsyg.2025.1565274

**Published:** 2026-01-13

**Authors:** Evgeniya Lukinova, Jeffrey C. Erlich

**Affiliations:** 1 NYU-ECNU Institute of Brain and Cognitive Science at NYU Shanghai, Shanghai, China; 2 NYU Shanghai, Shanghai, China; 3 N/LAB, University of Nottingham, Nottingham, United Kingdom; 4 Shanghai Key Laboratory of Brain Functional Genomics (Ministry of Education), East China Normal University, Shanghai, China; 5 Sainsbury Wellcome Center, University College London, London, United Kingdom

**Keywords:** delay discounting, chronic stress, cortisol, waiting, postponing

## Abstract

Stress is a normal part of our everyday lives, alerting us to changes in our environment and working as an early warning system. However, when stress is prolonged, it can become harmful. The deleterious effects of stress on brain function are well established: chronic stress significantly impairs cognitive function, reducing our ability to solve problems and regulate behavior. Therefore, it may lead to more challenges that can further exacerbate stress. An important class of decisions made under stress includes those between rewards delivered immediately and those delivered in the future. Not considering or devaluing future outcomes (delay discounting) can result in adverse outcomes, such as not buying health insurance, gambling, or drug use. To date, however, little is known about how chronic stress influences economic decisions that differ in the timing of outcome delivery. A handful of studies suggest that increased stress may lead to more impulsive choices in subjects of average socioeconomic status and stress levels. In this study, we addressed this gap by using a multi-session design to determine whether chronic stress measures (via questionnaires, saliva, and hair samples) are associated with economic choices across different time scales within subject (*N* = 34). We found that the degree to which people think it is worth waiting, i.e., an individual's discount factor, varied reliably over seconds but not over days in response to endogenous stress. These results are crucial for understanding the impact of stress in various contexts. For instance, fluctuations in individual stress responses to the environment may help explain variations in consumer behaviors, such as impulse purchases, even among healthy adults.

## 1 Introduction

Prolonged stress is known to have a negative influence on physical health (Harvey et al., 2003; Geronimus et al., 2010; Shchaslyvyi et al., 2024) and psychological well-being (Faravelli and Pallanti, 1989; Kessing et al., 2003; Hammen, 2005; Dixon and Overall, 2016; Fabbro et al., 2020). Negative effects of stress on behavior can be seen through a range of maladaptive behaviors, such as poor emotional control and social withdrawal. Still, the distinction between the effects of short-term (or acute) and long-term (or chronic) stress is fundamental to understanding how stress impacts decision-making quality (Lerner et al., 2012; Mullainathan and Shafir, 2013; Kandasamy et al., 2014; Haushofer and Fehr, 2014; Cohn et al., 2015; Riis-Vestergaard et al., 2018; Sarmiento et al., 2024). Many studies find no significant effect of acute stress on economic choices (Lempert et al., 2012; Sokol-Hessner et al., 2016; Forbes et al., 2024; Garcia Campos and Lempert, 2025). On the contrary, a controlled state of chronic stress in a laboratory experiment significantly increased risk aversion and led to steeper delay discounting (Kandasamy et al., 2014; Riis-Vestergaard et al., 2018). Field data also support a correlation between chronic stress and poor economic decisions. For example, chronically stressed individuals take on high-interest loans, have higher rates of substance abuse, display a lower willingness to take risks and forgo current income for larger future incomes (Mullainathan and Shafir, 2013; Haushofer and Fehr, 2014; Haushofer and Salicath, 2023). Moreover, stress may lead to maladaptive and potentially dangerous behaviors, making this study relevant to the prevention of substance dependence and various psychological disorders (Linsky and Straus, 1986; Lempert et al., 2012, 2018b; Lopez-Guzman et al., 2019; Knezevic et al., 2023).

In the animal literature, stress has been repeatedly linked to systematic shifts in decision strategies. Exposure to chronic and uncontrollable stress produces lasting neurobiological changes in hippocampal, prefrontal, and amygdala circuits, disrupting cognitive processes (Shors et al., 1992; McEwen and Sapolsky, 1995; Graham et al., 2010). Chronic stress in rats produces frontostriatal reorganization and a strong bias from goal-directed toward habitual, outcome-insensitive responding (Dias-Ferreira et al., 2009). Acute stress similarly affects higher cognition, biasing rats' cost-benefit evaluations (Shafiei et al., 2012). Acute fluctuations in stress hormones are also linked to coping behavior and prefrontal-midbrain interactions, as activation of this pathway shifts rats between active and passive coping modes and changes cortisol levels (Johnson et al., 2022). Research in primates suggests that individual differences in baseline cortisol predict whether animals “choke” or “thrive” under pressure (Sosnowski et al., 2022), mirroring rodent findings that stress hormones modulate effort-based choices and coping behaviors. These animal findings parallel human research, showing stress-related biases in decision-making (e.g., Lenow et al., 2017), which facilitates meaningful cross-species comparisons.

We define stress as the “organism's reaction to environmental demands exceeding its regulatory capacity” (Haushofer and Fehr, 2014). When a stressor is perceived, the hypothalamic-pituitary-adrenal-axis (HPA-axis) is activated and eventually releases cortisol (a steroid hormone, Schepers and Markus, 2015). Cortisol influences, regulates, or modulates many of the changes that occur in the body in response to stress. Hydrocortisone tablets work as a hormone replacement for a natural hormone called cortisol and are used in laboratory experiments to mimic the state of chronic stress (Kandasamy et al., 2014; Riis-Vestergaard et al., 2018).

Several common measures of chronic stress used in the extant literature include questionnaires (Goodman et al., 1998; Kopp et al., 2010; Raio et al., 2022; Guo et al., 2023), collecting heart rate and blood pressure (Gooding et al., 2015; Chalmers et al., 2021), hair (Karlén et al., 2011; Ceccato et al., 2015; Hakeem et al., 2025), and saliva samples (Pruessner et al., 2003; Inder et al., 2012; Kandasamy et al., 2014; Raio et al., 2020). Stress questionnaires can assess self-reported “stress” traits or measure perceived stress. Saliva samples provide a measurement of cortisol concentration at a single point in time (Hellhammer et al., 2009). Still, instead of tracking the peak of the diurnal cycle, it is also possible to observe cortisol levels over several days at the exact same time (Pruessner et al., 2003; Inder et al., 2012; Dietrich et al., 2013; González-Cabrera et al., 2014; Meggs et al., 2016), making it a better proxy for chronic stress (Lenow et al., 2017; Weckesser et al., 2019). Compared to other biological stress measures, hair samples offer certain advantages, including their ability to be stored and transported at room temperature and to reflect cortisol levels over the duration of hair growth (Karlén et al., 2011; Lawes et al., 2022; Kokka et al., 2023). Of course, all measures of stress are subject to some error. Having several measurements in this study and the ability to extract a few helps improve control and validation of the measurement system.

In this study, we aimed to address a fundamental gap in understanding how naturally occurring stress influences decision-making across different temporal contexts. While much research has demonstrated inconsistent or no effects of acute stress on how we evaluate alternatives, less is known about how endogenous stress (e.g., arising from daily life circumstances such as financial instability, relationship strain, or health concerns) shapes economic preferences over time. Time preferences, which involve trade-offs between a larger outcome received later and a smaller outcome received sooner, have been found to predict various outcomes, including academic achievement, income, and antisocial behavior (Golsteyn et al., 2014; Åkerlund et al., 2016; Exum et al., 2023). Yet, the majority of existing studies focus on decisions about outcomes delayed by days or weeks, neglecting choices that require active waiting for seconds, despite their ecological relevance and potential for cross-species comparisons. Our previous studies demonstrated strong correlations and stability in time preferences across seconds and days (Lukinova et al., 2019; Lukinova and Erlich, 2021), suggesting shared underlying mechanisms.

In this study, we explicitly tested whether the costs of waiting (short timescale) and postponing (long timescale), building upon the distinction made by (Paglieri 2013), share inter- and intra-individual variability with chronic stress levels, measured through both biological and self-report indices. By focusing on natural variation in stress within a healthy working population, our study complements acute stress induction paradigms by examining the effects of stress that accumulate over time, rather than those induced acutely in the laboratory. We chose a multi-session design to capture the dynamic relationship between stress and time preferences. Following the existing literature, we hypothesized that we would find a positive relationship between behavioral impulsivity and bio- or self-reported stress: an individual with a higher stress level would consider fewer alternatives in the future (Riis-Vestergaard et al., 2018; Wang et al., 2020). We also hypothesized that individual fluctuations in stress levels would be associated with similar trends in decision-making; that is, an increase in endogenous stress from one session to the next would predict more impulsive choices, an effect that has not yet been confirmed by experimental studies (Lempert et al., 2018a).

To the best of our knowledge, this is the first attempt to integrate a validated set of incentivized delay-discounting tasks across multiple time scales, biological stress markers (hair and salivary cortisol), and self-reported stress measures in a within-subject, multi-session experimental study involving a sample of healthy working adults.

First, we separately explored time preferences and measures of chronic stress. Second, we examined the similarity between distinct stress measures and delay discounting using correlation analysis. Third, we constructed predictive models by separating the effects of stress on within-subject and between-subject behavioral variability. For the within-subject analysis, we used the changes in biological stress markers over time to predict the changes in delay discounting over time (in seconds and in days, separately). For the between-subject analysis, we related the biological stress markers and self-reported stress measures to subjects' discount factors in seconds and in days. Finally, we assessed whether we could accurately classify subjects with similar behavior profiles by including their stress measures. We believe that using a unique set of stress measures alongside behavioral measures has enabled us to advance scholarship on how time preferences interact with hormonal and neuromodulatory systems.

## 2 Materials and methods

### 2.1 Participants

In this study, we recruited a total of 44 participants (26 women), including 15 participants from the New York University (NYU) Shanghai staff and 29 participants from the Chinese working population surrounding the NYU Shanghai and East China Normal University (ECNU) campuses. We pre-screened subjects before recruitment and excluded smokers, drinkers, and subjects taking medicine or suffering from acute or chronic hormonal dysregulation. Three participants withdrew after the first session, and the remaining 41 (23 female, *M*_*age*_ = 30) completed the entire experiment, with 34 participants having all stress measures. All of our participants shared similar socioeconomic characteristics (Supplementary Figure S1A). 31 participants (out of 41) had no siblings, 22 (out of 41) were unmarried, and 32 (out of 41) had no chronic illnesses.

The recruitment and experimental sessions for the study were conducted between April 2nd and October 20th, 2018. The study was approved by the Institutional Review Board (IRB) of NYU Shanghai. All subjects gave informed consent (written) before participation in the study. All research was performed in accordance with relevant guidelines and regulations.

### 2.2 Experimental tasks

**Figure 1:**
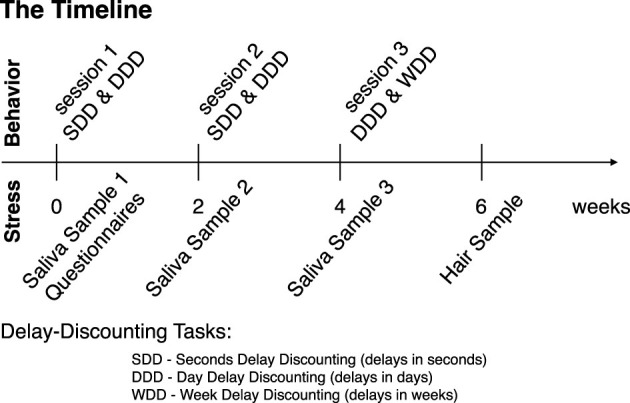
Experimental timeline. Each session took place every other week. During session 1, subjects provided the first saliva sample (by chewing on a synthetic swab), completed the questionnaires, and participated in seconds and day delay-discounting (SDD and DDD, respectively) tasks on computers, counterbalanced across subjects. During session 2, subjects provided a second saliva sample and participated in the same tasks as in session 1. During session 3, subjects provided the third saliva sample and participated in day delay and week delay tasks (DDD and WDD, respectively). Two weeks after session 3, subjects returned to provide the hair sample (hair strands with a total thickness of a toothpick and at least 3 cm in length, cut twice).

There were three behavioral task sessions in total (Figure 1). They were scheduled approximately every 2 weeks and took place in the NYU Shanghai Behavioral and Experimental Economics Laboratory, or the experimental room in the Geo Building at ECNU. Participants received a participation fee of 30 CNY ($4.3 USD) per session, plus up to an additional 70 CNY ($9.9 USD) per session based on their individual performance in the tasks. Each session consisted of a two-alternative choice task. During the first two sessions, subjects completed tasks involving delays measured in seconds and days (seconds delay discounting (SDD) and day delay discounting (DDD): 200 trials per task, Figure 1). During the third session, subjects participated in tasks with delays measured in days and weeks (DDD & week delay discounting (WDD): 100 trials per task, Figure 1).

We followed the same procedure for each delay-discounting task established in our previous works (Lukinova et al., 2019; Lukinova and Erlich, 2021). The main difference was that both the instructions and the screen stimuli were presented in Chinese. During the first two sessions, subjects experienced an alternating set of four tasks with the order counterbalanced. Therefore, subjects could experience one of the following sequences: SDD-DDD-SDD-DDD or DDD-SDD-DDD-SDD. During the third session, subjects completed two tasks in a counterbalanced order, i.e., DDD-WDD or WDD-DDD.

**Figure 2:**
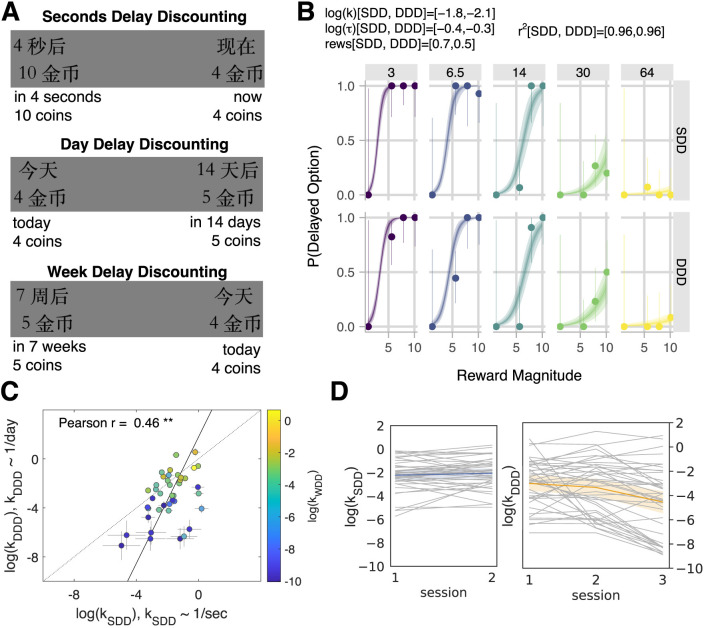
Comparison of model-based parameters across tasks: SDD (sessions 1 & 2 with delays in seconds), DDD (sessions 1, 2 & 3 with delays in days), and WDD (session 3 with delays in weeks). **(A)** Stimuli examples in the delay-discounting tasks through screenshots as presented to subjects in Chinese (Mandarin) and translated into English below. The “now/today” option appeared on the left or the right side of the screen, depending on the random draw. **(B)** Model fit of an example subject (for SDD & DDD in sessions 1 & 2 combined). In each panel, the marker and error bar indicated the mean and binomial confidence intervals of the subject's choices for that offer. The smooth ribbon indicated the BHM model fits (at 50, 80, and 99% credible intervals). At the top of the subject plot, we reported the mean parameter estimates for each task for that subject. We also listed the Bayesian *r*^2^ for each task. **(C)** Time preferences across time horizons. Each circle was one subject (*N* = 41). Discount factors were estimated in days but then converted to the units of the task. The error bars were the SD of the estimated coefficients. The solid line represented the perpendicular (or total) least squares (TLS) regression line (Huang et al., 2013). The dotted line was the unity line (*y* = *x*). The logs of discount factors (log(*k*)) in the seconds delay task (SDD, sessions 1 & 2, x-axis) were plotted against the logs of discount factors in the day delay task (DDD, sessions 1 & 2, y-axis). The color of the circles, aligned with the logs of discount factors in the week delay task (WDD, session 3), is shown in a color bar. Pearson correlation reported on the plot (*r* = 0.46, *p* = 0.003; ** for *p* < 0.01). **(D)** Stability of delay-discounting behavior across sessions in SDD (left) and DDD (right). Each subject's log(*k*) (y-axis) was plotted via a line plot (with the x-axis being an experimental session). The mean and 95% confidence interval were plotted using different colors.

In each trial of the SDD, subjects made a decision between the “now” and the “in *n* seconds” options (Figure 2A). If subjects chose a delayed option, they had to experience the delay immediately after making the choice. For example, they had to watch the clock for 30 s (if they chose the option “in 30 s”) until the screen disappeared, and then they could proceed to the next trial. The “now” reward was fixed at 4 coins; the “later” rewards used in the trials were 1, 2, 5, 8, and 10 coins (five in total), combined with delays to rewards: 3, 6.5, 14, 30, and 64 s (five in total). The two ‘smaller-later' options (1, 2) made up 10% of later options. Subjects accumulated coins, which were transferred at the end of the task to CNY, following the exchange rate provided in the instructions.

In contrast, for other delay-discounting tasks (with delays in days or weeks), subjects made a decision between the “today” and the “in *n* days/weeks” options (Figure 2A), saw their choice confirmation, and then proceeded to the next trial. The coins were not accumulated. The “today” reward was fixed at 4 coins; the “later” rewards used in the trials were the same as in SDD, with the same delay-to-reward values in days for DDD and in weeks for WDD. At the end of the session, a single trial was randomly selected, and the coins earned in that trial were converted to CNY using the exchange rate to determine the payment (balanced across tasks). If the subject chose the latter option in the randomly selected trial, the payment was transferred only after several days or weeks delay.

### 2.3 Stress measures

We used three questionnaires to measure perceived stress. The 10-item version of the Perceived Stress Scale (PSS, Cohen et al., 1983) was used to assess an individual's subjective appraisal of particular life events/situations as being unpredictable, uncontrollable, and/or overloaded. On a 5-point Likert scale ranging from 0 “never” to 4 “very often,” participants rated how often in the previous month they felt or thought as described in 10 examples (aggregated in the “pss” variable). The Brief Encounter Psychosocial Instrument (BEPSI, Frank and Zyzanski, 1988) usually consists of six questions. We omitted the first open-ended question and used the remaining five closed-ended questions. Subjects were asked to respond yes or no to each item and, if yes, to rate the impact of these stressors on a scale of 1 to 10 (aggregated in the “bepsi” variable). The Social Readjustment Rating Scale (SRRS, Holmes and Rahe, 1967) was used to identify major stressful life events. Each of the 43 stressful life events was assigned a Life Change Unit (LCU) based on how traumatic it was perceived to be by a large sample of participants. The total was then calculated by summing all LCUs for the events a subject experienced (”lcu” variable). Thus, in all questionnaires, higher scores were associated with higher levels of perceived stress. All questionnaires were administered in Chinese and completed after the first saliva sample was provided.

In order to obtain a biological measure of participant's stress levels, we collected a saliva sample from each participant at the beginning of each session. In healthy individuals, cortisol levels peak in the early morning and then gradually decrease. To help overcome this challenge, most studies collect multiple human specimen samples from waking until sleep or gather samples at the same times over several days. For each participant, the collection time remained the same across three sessions (representing chronic stress, as in Pruessner et al., 2003; González-Cabrera et al., 2014). Therefore, we could compare salivary cortisol levels in sessions 2 and 3 (ls2 and ls3, respectively) to the baseline (ls1 in session 1) and attribute these deltas to the changes from baseline cortisol levels. Saliva samples were collected using the Salivette system. The Salivette systems (Sarstedt, Shanghai) came with synthetic swabs. Subjects were instructed to avoid eating, drinking, and/or brushing their teeth for at least 1 h before sampling and to rinse their mouth 10 min before arriving at the experimental facility. Before each experimental session, the experimenter asked the subject to put the swab in their mouth, chew it for 1 min, and then transfer it into the tube. All subjects provided three saliva samples of sufficient quality. The samples were stored on ice until the end of the experimental day, then centrifuged and kept at –20°C until analysis at the NYU Shanghai Molecular Biology Lab. On the day of analysis, we measured salivary cortisol using an enzyme-linked immunosorbent assay (ELISA) (IBL International, Germany). Saliva samples were assayed in duplicate (40 * 2 samples per ELISA kit) and measured according to the instructions for each kit. The controls were within expected bounds.

**Figure 3:**
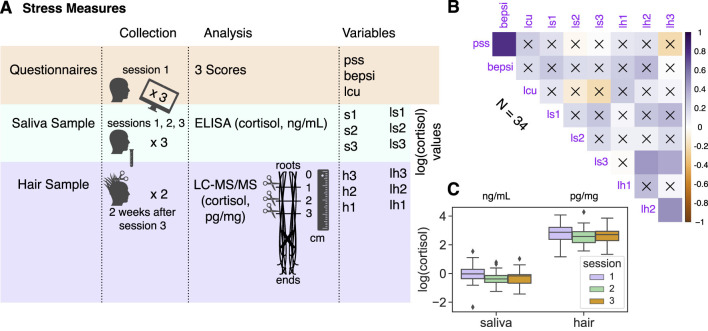
**(A)** Stress measures collection, analysis, and related variables (see details in Section Materials and methods). Subjects filled in three questionnaires in session 1: Perceived Stress Scale (PSS, Cohen et al., 1983); the Brief Encounter Psychosocial Instrument (BEPSI, Frank and Zyzanski, 1988); and the Social Readjustment Rating Scale (SRRS, Holmes and Rahe, 1967) to get life change units. Also, subjects provided three saliva samples in total, one at the beginning of each experimental session. Finally, most of the subjects returned 2 weeks after session 3 to provide a hair sample (hair strands with toothpick thickness at least 3 cm in length cut twice were merged from the cut and then divided into three 1 cm segments reflecting the chronic stress two months, one month prior to, and at the month of collection). **(B)** Correlation plot across all stress measures. The color intensity (vertical color bar) indicated the correlation coefficient *r*. The insignificant correlations, e.g., where *p* ≥ 0.05, were indicated with an “x”. **(C)** Boxplots of log(cortisol) for saliva and hair samples across three experimental sessions.

Two weeks after all three experimental sessions were finished by the subjects, we also collected their hair samples to measure chronic stress levels. A total of 36 subjects returned to provide hair samples. Hair samples were collected by cutting and joining two strands of hair at the cut from the posterior vertex area, each about the thickness of a toothpick. All samples were stored in aluminum foil at room temperature. LC-MS/MS (liquid chromatography with tandem mass spectrometry) atmospheric pressure chemical ionization (APCI, MRM mode) was used to analyze cortisol in hair samples. The analysis was conducted by Dr. Huihua Deng's lab at Southeast University in China using their standard approach (Chen et al., 2013). Hair strands longer than 3 cm were cut as close to the scalp as possible and divided into three 1 cm segments (analyzing each 1-centimeter segment separately to assess stress over the last three months). All segmented samples were labeled as SXxx-01 to SXxx-03 from bottom (closest to the scalp) to top, which later became the lh3-lh1 variables, respectively (Figure 3A). A total of 36 times 3, which equals 108 samples, were analyzed. The recovery rate was within 90-105%, and intra-day and inter-day coefficients of variation were below 10%, which satisfied measurement requirement.

### 2.4 Analysis

#### 2.4.1 Fitting economic choices

Subjects' discount factors were estimated by fitting their choices (24,277 choices across all experimental sessions) with a Bayesian hierarchical model (BHM) of hyperbolic discounting with decision noise and a reward scaling parameter, ‘rews' that scaled the delayed reward per unit. Bayesian hierarchical modeling enables pooling data across subjects while distinguishing between two delay-discounting tasks within each session and accounting for subject-specific differences. One of its advantages is also the ability to estimate posterior distributions rather than obtaining only point estimates of the parameters. We did not compare different functional forms and followed our previous study by using the softmax-hyperbolic model (Lukinova et al., 2019). Compared to the exponential model (Samuelson, 1937) of time discounting, which has a straightforward economic meaning – a constant probability of loss of reward per waiting time, the hyperbolic model (Mazur, 1987), according to scholarship, more accurately describes how individuals discount future rewards. At the same time, a shift-invariant SoftMax rule is commonly used in the field to transform the subjective utilities of the sooner and later offers into a probability of choosing the later offer.

The main “reward scale” model (*M*_6*p*, 3*s*_) had six population-level parameters (log(*k*), decision noise, log(τ), and the reward scale parameter, rews, for each of the two delay-discounting tasks per session) and 3 parameters per subject: e.g., for session 1 log(*k*_*SDD*1_), log(*k*_*DDD*1_) and log(τ).


n_chose_later | trials (n_trials) ~ ( exp (rews)
* later_reward / (1 + exp(logk) * dl)
- sooner_reward) / exp (logtau),
logk ~ unit + (unit|subjid),
logtau ~ unit + (1|subjid),
rews ~ unit


where dl was delay, later_reward was the later reward, sooner_reward was the sooner reward; logk was the natural logarithm of the discounting parameter *k* and logtau (log(τ)) was the natural logarithm of the decision noise. In the binomial specification, the data were grouped and summarized by distinct trial types, where n_chose_later was the count of choices when the later reward was selected, and n_trials was the number of trials for a particular trial type.

Based on the 10-fold cross-validation criteria, the main model was preferred over models with fewer parameters (Supplementary Table S1). The main model was estimated after converting all delays to days (resulting in the same fits as the fits in the units of the task) with the “brms” package in R (Bürkner, 2017) that allows one to do BHM of nonlinear multilevel models in Stan (Carpenter et al., 2016) with the standard R formula syntax.

#### 2.4.2 Making comparisons

Permutation tests for differences between the means of two groups were performed by shuffling the group labels and computing the mean between the shuffled groups 10,000 times. This generated a null distribution, which was used to estimate the probability of observing the true difference between groups (bootmean in https://github.com/erlichlab/elutils). The visualizations of the correlation matrices were done via the R package “corrplot” (Friendly, 2002). Whenever multiple hypotheses were tested simultaneously (e.g., multiple correlations were run between the discount factor and the stress variables), we used the Bonferroni multiple comparison correction with an α-level of α/*n*, where *n* was the number of hypotheses.

#### 2.4.3 Predictive modeling

We used Lasso, Ridge, and Elastic Net cross-validated (5-fold) regressions with standardized data to predict discount factors and their change using stress variables (within-subject and between-subject analyses). In particular, the “sklearn” Python library classes “sklearn.linear_model.LassoCV,” “RidgeCV,” and “ElasticNetCV” were used, respectively. We tried an array of α values (penalties: 0.001, 0.01, 0.1, 1). Once the optimal penalty parameter was determined through cross-validation, the model was refitted using the entire training set. We chose these models due to their ability to address issues of overfitting and multicollinearity. They achieve this by adding a penalty term to the cost function that shrinks the magnitude of the estimated coefficients, resulting in improved generalization performance and more robust predictions. Ridge regression (where regularization is given by the l2-norm) outperformed other models on the coefficient of determination and may be especially relevant for our data, given correlations among input features and a small dataset.

**Figure 4:**
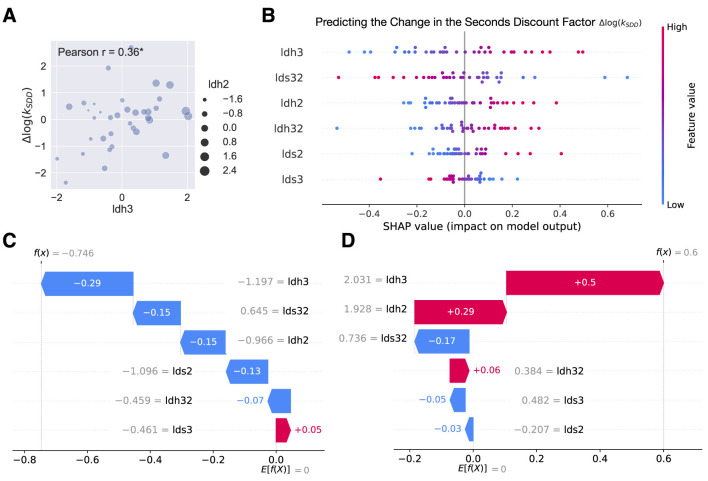
Within-subject analysis. **(A)** Scatterplot of change in hair cortisol (Δlh3, x-axis) and change in seconds discount factor (Δlog(*k*_*SDD*_), y-axis). The size of the dots was aligned with Δlh2. Pearson's *r* was reported on the plot (*r* = 0.36, *p* = 0.033; * for *p* < 0.05). **(B)** SHAP summary plot (beeswarm) that explained the within-subject Ridge regression predictions of the change in the seconds delay discount factor (log(*k*_*SDD*2_)−log(*k*_*SDD*1_)). The independent variables (features) were displayed in descending order by importance. Each subject was represented by a single dot on each variable row. The x position of the dot was determined by the SHAP value of that feature, i.e., showing whether the effect of that value was associated with a higher or lower prediction. Color was used to display the original value of a feature. **(C, D)** Examples of two extreme subjects' predictions. The waterfall plot explained predictions for individual subjects, i.e., it showed how the positive (red) or negative (blue) contribution of each feature moved the value from the expected change in the seconds delay discounting across sessions to the value of this subject's prediction. Numbers on the arrows or in color indicated individual SHAP values; numbers in gray represented the standardized values of the stress variables for the subject under consideration.

The Shapley Additive exPlanations (SHAP, using the Python library “shap”) were crucial in explaining the predictions of our best Ridge regressions. Following Lundberg and Lee (2017), we imagined that the regression model inputs are players and the model predictions are players' payouts; SHAP then provided the contribution of each player to the game. Not only can combined SHAP values show which features are important and the direction of correlation with the prediction (Figure 4B), but also for each individual prediction (e.g., prediction of the change in subject's seconds discount factor), individual SHAP values detail the contribution of each feature and show how each feature moves the value from the expected value of the output feature (dependent variable) to this particular subject's prediction (e.g., for two extreme subjects Figures 4C, D).

Finally, to predict the classes of subjects who increased or decreased their patience in the delay task across sessions, we used three commonly used classifiers: logistic regression, decision tree, and random forest (all implemented in the Python library “sklearn”). The “tree” non-parametric models were considered to allow for complex nonlinear relationships between input features and the output. We implemented 5-fold stratified cross-validation and compared the models using balanced accuracy and the area under the receiver operating characteristic curve (AUC-ROC) due to class imbalance (out of 34 subjects, 27 showed increased patience, while only seven subjects remained at the same level or decreased in patience). Stratified cross-validation ensured the model was trained and tested on representative subsets of the data, thereby reducing the risk of overfitting and enhancing generalization performance. The logistic regression model outperformed other models in predicting the majority class (by accuracy) but was unable to accurately predict the minority class (as indicated by the confusion matrices).

### 2.5 Software

The code for the delay-discounting task was written in Python using the “PsychoPy” toolbox (version 1.83.04; Peirce, 2007) available at https://www.github.com/erlichlab/gpstress/src/task. All analyses and statistics were conducted using MATLAB (version 9.3 or higher, The MathWorks, MA), R (version 3.6.1 or higher, R Foundation for Statistical Computing, Vienna, Austria), or Python (version 3.9.13).

## 3 Results

### 3.1 Delay-discounting behavior is stable and reliable

In each trial of the delay-discounting task, subjects made a decision between the sooner and later options (Figure 2A). The three tasks differed in the delay-to-reward, measured in seconds (SDD), days (DDD), or weeks (WDD). We fit subjects' choices using a Bayesian hierarchical model (BHM) of hyperbolic discounting, following our previously validated methodology (Lukinova et al., 2019), to elicit time preferences. Thus, the subject fits are comprised of mean estimates of log(*k*) – logarithm of the delay-discounting factor (discount factor), log(τ) – logarithm of the decision noise and reward scale parameters for each subject per experimental session, and two delay-discounting tasks within it (Figure 2B).

The subject fits and correlations between fits across sessions and tasks were robust to the model choice (Supplementary Table S2). The subjects' choices were well fit by the model as assessed using Bayesian *r*^2^ and reported in each of the subject delay plots (Figure 2B for an example subject fit; see Supplementary material for all subject plots in Supplementary Figures S2, S3 and posteriors in Supplementary Figure S4). Consistent with previous scholarship (Lukinova et al., 2019; Lukinova and Erlich, 2021), we found that time preferences measured through discount factors showed strong correlations across different time horizons: between days and seconds (Pearson *r* = 0.46, *p* = 0.003, Figure 2C) and between days and weeks (Pearson *r* = 0.96, *p* < 0.001). Across sessions, only log(*k*) in DDD was significantly different between session 3 and the first two sessions according to permutation tests (e.g., log(*k*): *M*_*s*1_ = −3.002 & *M*_*s*3_ = −4.534, *p* < 0.001, Section Materials and methods). On the contrary, the economic behavior in both SDD and DDD tasks between session 1 and session 2 was stable (permutation tests for log(*k*) difference were not significant, *p* > 0.1, also in Figure 2D). Subjects who increased their patience over the gained experience with the task (i.e., for whom log(*k*) in session 3 was smaller than the average between sessions 1 & 2) were further explored.

### 3.2 Chronic stress variables convey distinct meanings

Rather than inducing stress experimentally, we focused on endogenous variations in subjects' stress levels (Figure 3, Section Materials and methods). For each subject we collected the base level of perceived stress using three questionnaires (forming “pss,” “bepsi,” and “lcu” variables) and biomarkers of stress via three saliva samples (log-transformed cortisol levels: ls1-ls3) and one hair sample divided into three 1 cm segments (log-transformed cortisol levels: lh1, lh2, and lh3 for the stress level two months before collection, one month before, and at the month of collection, respectively, following Gow et al., 2010).

According to the literature, we expected a significant medium-level correlation between hair and saliva measures around *r* = 0.4 (Vanaelst et al., 2012; van Holland et al., 2012; Zhang et al., 2018; Weckesser et al., 2019) and a low-level to no correlation between biological measures and questionnaires (Vanaelst et al., 2012; Stalder et al., 2017; Prado-Gascó et al., 2019). Some scholars suggest log-transforming cortisol values to better approximate a Gaussian distribution across subjects (Lenow et al., 2017; Lempert et al., 2018a). Indeed, we decided it is reasonable to modify all of our cortisol values based on the distributions before and after log-transformation (Supplementary Figure S5).

In the correlation analysis, which combined all stress measures, cortisol levels were not associated with questionnaire scores. Overall, we found stronger correlations within stress measure groups (which may validate stress measures in general) than across them (Figure 3B). Still, as expected, aggregated salivary cortisol across three samples (ls123) was positively correlated with two hair cortisol samples (ls123 vs. lh2: Pearson *r* = 0.38, *p* = 0.024; ls123 vs. lh3: Pearson *r* = 0.35, *p* = 0.039; as in Weckesser et al., 2019). With the multiple comparison correction (8 comparisons per stress variable: α-level 0.05/8), only positive correlations between lh2 and lh3 (Pearson *r* = 0.46, *p* = 0.005) and PSS and BEPSI questionnaires (Pearson *r* = 0.78, *p* < 0.001) remained significant. Stronger correlations within the stress measure groups were further confirmed with a principal component analysis (PCA: Supplementary Figure S6 and Supplementary Table S4): the second principal component, along the nine stress vectors (all stress variables), distinguished between human biosamples (hair and saliva) and questionnaires, whereas the third principal component distinguished between hair and saliva stress measures. Salivary (ng/mL) and hair (pg/mg) cortisol concentrations remained within consistent ranges, respectively, across experimental sessions (Figure 3C). According to permutation tests, hair samples were not significantly different, while ls1 (session 1 saliva sample) was significantly higher than that for the other sessions (e.g., *M*_*s*1_ = −0.047 & *M*_*s*3_ = −0.330, *p* = 0.011).

Although gender differences in cortisol and other neuroendocrine markers have been documented (Takahashi, 2004; Barel et al., 2017), alongside menstrual and contraceptive effects (Lempert et al., 2012; Stalder et al., 2017), permutation tests revealed no significant gender effects on stress variables in our sample (*p* > 0.09). Additionally, by visually assessing the differences through the first three principal components and their respective 2D planes, we found that the gender ellipses, as well as the data, highly overlapped (Supplementary Figure S6).

### 3.3 Stress variables were selectively predictive of the seconds discount factor

It is important to distinguish between the effects of endogenous variation in stress on within-subject and on between-subject variability in choice preferences. Both can be examined using the stress measures and the economic tasks in this study.

First, given repeated economic tasks and biological measures across the experimental sessions/months, we could examine whether within-subject changes in behavior were related to changes in stress levels. In particular, we wanted to explore subtle deviations from the base level (given that both discount factors and cortisol measures were stable on average in Figures 2D, 3C). Second, given that the ranges of stress measures in our study matched those of the healthy population (Supplementary Table S3), we explored whether stress measures per session/month shared intersubject (between-subject) variance with economic behavior. In other words, can we predict subjects' behavior in delay-discounting tasks by using a combination of stress measures over time?

Correlational analysis revealed a significant positive correlation between the change in the seconds delay discount factor (log(*k*_*SDD*2_)−log(*k*_*SDD*1_)) and hair cortisol change (Δlh3 = lh3 - lh1, Pearson *r* = 0.36, *p* = 0.033, Figure 4A, no longer significant if we used the Bonferroni multiple comparisons correction; other correlations in Supplementary Table S5). Then we ran a conventional set of regression models (including Lasso, Ridge, and Elastic Net regressions) with 5-fold cross-validation and standardized data (Section Materials and methods). Ridge regression outperformed other models, and the only model that had the coefficient of determination (*R*^2^) bigger than 0.2 was predicting the change in the seconds delay discount factor (Table 1 and Figures 4B–D). We used the SHapley Additive exPlanations (SHAP) to interpret the model predictions (Section Materials and methods). According to the SHAP summary plot (Figure 4B) first three features in the order of importance were Δlh3, Δls32 = ls3 – ls2, and Δlh2 = lh2 - lh1. The hair cortisol change had the largest positive impact on the prediction of the change in seconds discount factor (consistent with our hypothesis). We decided to unpack the SHAP summary further by focusing on the two example subjects at the extremes; the same three stress variables appear as the top predictors. For one of them, the 2 standard deviation increase in stress level across months (Δlh3 = 2.031) was the single main contributor to the prediction of increased impulsivity (by 0.5 standard deviations, Figure 4D) in the seconds delay task; for the other one, the 1 standard deviation decrease in stress level across months (Δlh3 = –1.197) was the main contributor to the prediction of increased patience (by 0.29 standard deviations, Figure 4C) in the seconds delay task.

**Table 1:** Ridge regression comparison.

**Within-subject *N* = 36**	**log(*k*_*SDD*2_) −log(*k*_*SDD*1_)**	**log(*k*_*DDD*2_) −log(*k*_*DDD*1_)**	**log(*k*_*DDD*3_) −log(*k*_*DDD*1_)**	**log(*k*_*DDD*3_) −log(*k*_*DDD*2_)**	
*R* ^2^	0.25	0.13	0.06	0.10	
**Between-subject** ***N*** **= 34**	**log(*k*_*SDD*1_)**	**log(*k*_*SDD*2_)**	**log(*k*_*DDD*1_)**	**log(*k*_*DDD*2_)**	**log(*k*_*DDD*3_)**
*R* ^2^	0.50	0.24	0.18	0.06	0.07

**Figure 5:**
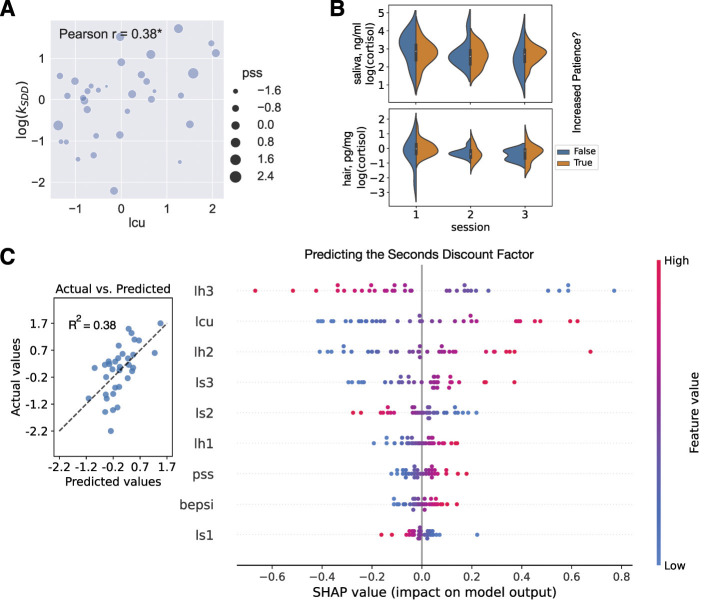
Between-subject analysis. **(A)** Scatterplot of life change units (lcu, x-axis) and seconds discount factor averaged across sessions (log(*k*_*SDD*_), y-axis). The size of the dots reflected another self-reported stress measure, the Perceived Stress Scale (pss). Pearson's *r* was reported on the plot (*r* = 0.38, *p* = 0.029; * for *p* < 0.05). **(B)** Violin plot visualizing no significant differences between log(cortisol) in saliva and hair samples between subjects who increased their patience in the day delay task in session 3 and those who did not. **(C)** Cross-validated predictions from the between-subject Ridge regression with the following dependent variable: log(*k*_*SDD*_) = (log(*k*_*SDD*1_)+log(*k*_*SDD*2_))/2. In the left subplot, the predicted values (x-axis) were plotted against the actual values (y-axis). The coefficient of determination was shown on the plot. The dashed line was the best-fit line. In the right subplot, the SHAP summary plot (beeswarm) explained the between-subject Ridge regression predictions. The independent variables (features) were displayed in descending order by importance. Each subject was represented by a single dot on each variable row. The x position of the dot was determined by the SHAP value of that feature, i.e., showing whether the effect of that value was associated with a higher or lower prediction. Color was used to display the original value of a feature.

Each column identified the dependent variable (the target). The within-subject models included six independent variables (features): Δls2, Δls3, Δls32, Δlh2, Δlh3, Δlh32. The between-subject models included nine independent variables (features): pss, bepsi, lcu, ls1, ls2, ls3, lh1, lh2, lh3. All variables were standardized before their use in the models. Ridge regressions were set up with the use of 5-fold cross validation and a search through parameter space for the best α from (0.001, 0.01, 0.1, 1, the penalty term in the Ridge regression). In Figure 5 the plots showed the results of predicting the seconds discount factor averaged across sessions (i.e., the dependent variable log(*k*_*SDD*_) = (log(*k*_*SDD*1_)+log(*k*_*SDD*2_))/2). This model had *R*^2^ = 0.38.

In the between-subject analysis, the average seconds delay discount factor correlated with the life change units (log(*k*_*SDD*_) vs lcu: Pearson *r* = 0.38, *p* = 0.029), and the seconds delay discount factor in session 1 correlated with the second principal component (log(*k*_*SDD*1_) vs PC2: Pearson *r* = 0.52, *p* = 0.002, remaining significant after correction at the α-level of 0.05/13), where PC2 was formed along the nine stress vectors: three saliva, three hair, and three questionnaires, representing a direction that separated human bio samples and self-reported measures (Supplementary Figure S6; further PCA details and other correlations in Supplementary material).

As in the within-subject analysis, Ridge regression outperformed other models, with the coefficient of determination (*R*^2^) exceeding 0.2 for models predicting the seconds delay discount factor (Table 1 and Figure 5C). SHAP for the between-subject analysis highlighted the importance of hair stress measures, with lh3, representing the stress level during the month of sample collection, as the key negative predictor of the discount factor. Additionally, the stress level the month before the collection (lh2) provided one of the largest positive contributions to predicting the seconds discount factor. Only the latter finding was expected (given our hypothesis derived from the literature) and intuitive: the higher the stress level, the higher the seconds discount factor relative to the average (i.e., more impulsive, Figure 5C). Another important feature in the between-subject regression (and in the two separate regressions with the seconds discount factor in sessions 1 & 2 as the dependent variable) is the life change units (lcu) that gave the biggest positive contribution to the seconds discount factor: the more traumatic experiences a subject experienced (higher lcu), the more the model predicted they discounted in seconds (higher discount factor was associated with increased impulsivity, again as per our hypothesis, Figure 5A).

Finally, we investigated whether we could classify subjects who increased their patience over the gained experience with the day delay task, based on their stress profile. There were 27 such subjects, as opposed to 7 subjects whose discount coefficient stayed at the same level or increased (decrease in patience). According to three basic classifiers (logistic regression, decision tree, and random forest, with the use of 5-fold stratified cross-validation; Section Materials and methods), the stress variables were not helpful to dissociate between increased and decreased patience (with overlapping distributions of stress supporting this result in Figure 5B). For example, the logistic regression model was able to predict the majority class (increase in patience) well with 79.5% accuracy but did not predict the minority class at all, resulting in a balanced accuracy of 50%. Therefore, temporal context, or the fact that the day delay task was paired with the week delay task in session 3 (as opposed to the seconds delay task), could be a more plausible explanation.

## 4 Discussion

In our study, biological stress measures were moderately correlated with each other, as reported in the literature, further validating the stress measurement system (Vanaelst et al., 2012; van Holland et al., 2012; Zhang et al., 2018; Weckesser et al., 2019). However, overall, we found stronger relationships within stress modalities than across them (also evident in the PCA analysis, Supplementary material). This provides further evidence that self-reported stress, salivary cortisol, and hair cortisol may influence decision making by operating through distinct channels.

The multi-session within-subject design enabled us to find effects of biological stress markers and their changes over time both on within-subject and between-subject behavioral variability. The strongest result we found for within-subject variability was that increases in cortisol levels were associated with increased impulsivity in the seconds delay task across experimental sessions, consistent with our hypothesis. However, the underlying mechanism explaining the positive effect of change in hair cortisol (Δlh3) but the negative effect of change in saliva cortisol (Δls32) remained unclear. The timing of the comparison was slightly different: a 1-month delta for the hair and a 2-week delta for the saliva, but we would not speculate further on this. In the between-subject analysis, although hair stress level was among the important regressors predicting subjects' time preference over seconds, the direction of influence for stress in the month of sample collection (lh3) compared to stress in the month prior to the collection (lh2) was directly opposite. Only life change units contributed to the prediction in the expected manner: more stress was associated with higher impulsivity in seconds, as per our hypothesis derived from previous studies. Additionally, models that captured behavior from the seconds delay discounting task did relatively better than those using a traditional delay-discounting task. The seconds delay discounting task was the only one where all trials contributed to both total profit and total waiting time, whereas in the longer-delay tasks, the outcome depended on a single random trial. We found that behavioral responses to endogenous stress were stronger in the experiential task, in which participants experienced waiting for all selected delays followed by immediate reward delivery as coins, than in tasks in which rewards were postponed to either the end of the session or a number of days specified in the randomly selected trial.

Previous research acknowledges both linear and curvilinear relationships between changes in cortisol as a physiological response to stress and behavior (Goldfarb et al., 2017; Maier et al., 2015; Luksys and Sandi, 2011; Lempert et al., 2018a). We did not find any curvilinear relationships between stress variables and model-free behavioral measures in a separate analysis (Supplementary material). This might be because tests for curvilinear relationships included only a small subset of participants, those for whom there was an increase in biological stress levels relative to baseline. In fact, SHAP values do not capture nonlinear relationships in our regression analysis and assume that the model is additive, meaning that each feature's contribution is independent of the other features, an assumption that might not hold. It is important to note that SHAP values derived from the Ridge regression model do not establish causal mechanisms; they show how each independent variable contributes to the model's prediction, rather than how the independent variables affect the dependent variable.

Among self-reported stress proxies, the life change units were positively correlated to the seconds discount factor and negatively correlated to the proportion of later choices in the seconds delay task. It is worth noting that the Life Change Unit is a measure of the real traumatic experience of subjects in a particular event, compared to two other questionnaires that ask for feelings about (The Brief Encounter Psychosocial Instrument) or subjective appraisal of (Perceived Stress Scale) particular life events or situations. Previous scholarship has found a robust relationship between stressful life events and increased negative health outcomes; however, only a few studies have investigated the relationship between life change units and decision making (Wang et al., 2020). Although we found that those with more traumatic experiences discount more, as expected, it might be only true for those with lower cognitive speed (Friedel, 2017). Lower levels of cognitive speed could worsen the conversion from seconds to days when subjects are presented with choices in the seconds delay task or diminish their ability to wait in real time. We did not include any measures of cognitive speed in this study to test this explanation.

In our study design, several limitations need to be acknowledged. First, the design lacked control tasks necessary to disentangle whether experiential waiting or financial consequences mediated stress-behavior associations. Fully isolating these factors would have required hypothetical variants of both short- and long-delay tasks, without experiential waiting or profit implications, thereby considerably increasing experimental complexity and participant burden. Second, delay-discounting tasks were not measured an equal number of times, which may have affected our results. This was due to both the desire to replicate previous behavioral results (Lukinova et al., 2019) in a general population group and to test more economic tasks within the same subjects. Third, questionnaires were not carried out repeatedly. Having questionnaires that reflect the self-reported stress in the within-subject regression might indicate new relationships between subtle changes in stress and behavior. Finally, our study had a relatively small sample size and experienced some attrition (17%), resulting in limited statistical power to detect significant effects. The limitations listed should be taken into consideration when interpreting the results of this study.

Our study did not induce stress in participants but rather examined cortisol levels, their changes, and fluctuations from the baseline, which may be due to individual responses to the environment. Observed variations likely reflect idiosyncratic neuroendocrine responses to environmental stimuli, representing a relatively novel methodological framework. Previously, in control groups, random fluctuations of cortisol did not produce the same behavioral patterns (Lempert et al., 2018a). However, the majority of previous studies were using saliva measures (Takahashi, 2004; Linz et al., 2018). Most of our significant relationships between stress and behavior involved the hair cortisol measure. We conclude that, for subtle behavioral changes resulting from random cortisol fluctuations, hair stress measures may be more reliable.

Measuring and administering hormones is a complex task because hormone levels vary throughout the day and month. For example, uncontrollable stressors tend to produce a higher overall daily cortisol release compared to controllable stressors – higher-than-normal morning cortisol (Miller et al., 2007). As a result, many studies involving hormones and decision making produce conflicting results or fail to be replicated, and therefore, they are unable to advance the current state of the discipline. To the best of our knowledge, this is the first study to combine self-reported stress, biological stress, and delay-discounting tasks across time horizons. Most of the significant relationships observed were between endogenous variation in hair cortisol and the seconds delay task, which had never been studied together. Some of our main results support the hypothesis in the literature that increased stress leads to steeper discounting. Still, we found that endogenous subclinical cortisol variation had only a weak correlation with economic preferences. The strongest links we found were that within-subject increases in stress levels led to increases in discounting, and higher stress levels between-subject were associated with higher discounting, both in the seconds delay task.

Additional multitask and multistress studies with higher *N* are required to establish the effects reported in this paper. An accurate understanding of the biological processes that lead people to discount future payoffs is still tenuous at best. Unraveling the mechanisms by which chronic stress spreads unfavorable effects to behavior is of critical importance to policymakers and of strong interest to the general public.

## Data Availability

The datasets presented in this study can be found in online repositories. The names of the repository/repositories and accession number(s) can be found below: https://doi.org/10.5281/zenodo.8192007.
